# Retracted: Recent Trends of Polymer Mediated Liposomal Gene Delivery System

**DOI:** 10.1155/2019/8195729

**Published:** 2019-08-08

**Authors:** BioMed Research International

BioMed Research International has retracted the article titled “Recent Trends of Polymer Mediated Liposomal Gene Delivery System” [1]. The article was found to contain a substantial amount of material from a number of published articles, including the following sources:

(i) L. E. van Vlerken, T. K. Vyas, and M. M. Amiji, “Poly(ethylene glycol)-Modified Nanocarriers for Tumor-Targeted and Intracellular Delivery,” Pharmaceutical Research, vol. 24, no. 8, pp. 1405–1414, 2007, https://doi.org/10.1007/s11095-007-9284-6 [2] (cited as [98]).

(ii) Daniel A. Balazs and WT. Godbey, “Liposomes for Use in Gene Delivery,” Journal of Drug Delivery, vol. 2011, Article ID 326497, 12 pages, 2011, https://doi.org/10.1155/2011/326497 [3] (not cited).

(iii) Vladimir P. Torchilin, “Recent Advances with Liposomes as Pharmaceutical Carriers,” Nature Reviews Drug Discovery, vol. 4, no. 2, pp. 145–160, 2005, https://doi.org/10.1038/nrd1632 [4] (cited as [35]).

(iv) Arcadio Chonn and Pieter R Cullis, “Recent Advances in Liposome Technologies and Their Applications for Systemic Gene Delivery,” Advanced Drug Delivery Reviews, 1998, https://doi.org/10.1016/S0169-409X(97)00108-7 [5] (not cited).

(v) Marika Ruponen, Seppo Ylä-Herttuala, and Arto Urtti, “Interactions of Polymeric and Liposomal Gene Delivery Systems with Extracellular Glycosaminoglycans: Physicochemical and Transfection Studies,” Biochimica et Biophysica Acta: Biomembranes, vol. 1415, no. 2, pp. 331–341, 1999, https://doi.org/10.1016/S0005-2736(98)00199-0 [6] (cited as [138]).

The authors do not agree to the retraction.
